# Digital Materiality and Doing Map History

**DOI:** 10.1080/03085694.2025.2567130

**Published:** 2025-12-25

**Authors:** Philip Jagessar

**Affiliations:** Kings College London

A concern for the material, as readers of *Imago Mundi* will know, is part of what defines map history as an epistemic community.^1^ Of course, map historians recognise—and have been at the forefront of arguing—that maps are more-than-artefacts and mapping is more-than-representational but there is a shared understanding that what we directly (or indirectly) study has (or had) a tangible and traceable existence in shaping what gets collectively called the ‘history of mapping.’^2^ It remains an expected rite of research passage for many doctoral students and early career map historians to visit ‘the archive’ and lay out a map(s) or open an atlas, building a sensory and experiential understanding of the material being examined. Further, despite priding ourselves on our objectivity, if we as a diverse community of map historians are honest, the relationship that we develop with maps or related materials we study is often deeply affective.

Nevertheless, a significant concern for the discipline at present is how the digitisation of existing collections and born-digital mapping will shape how critical map history is done.^3^ Less well discussed has been the challenge of making map historical approaches relevant to future generations of emerging scholars who were themselves ‘born digital’ and will primarily have had exposure to maps that are the product of lines of code and interfaced through apps, websites and software. In this *Forum* piece, I reflect on my work with a historical map collection that has been entirely digitised: the League of Nations (LON) map collection, which forms part of the larger UN (United Nations) Library and Archives in Geneva. Significantly, the UN Archives *only* allows users to interact with the collection through a digital interface, conditioning both the collection and its users to ‘become-digital’ and blurring the line between both the material and the virtual and the archivist and historian.^4^ This policy remains unusual for now, as most digitised collections still allow users to see physical maps if they come to the collections in person. It nonetheless raises important questions, particularly as such policies become not only more common but quite likely the normal way of doing map history.

The intensification of archival and collection digitisation in recent decades means that all stages of academic research is increasingly mediated through the digital.^5^ Well-known public digitisation projects have been undertaken at the Library of Congress, the Bibliothèque nationale de France, and the University of Wisconsin-Milwaukee (AGSL Digital Map Collection), among others. The gold standard digitised map collection for many is the David Rumsey Historical Map Collection, which began as a private endeavour. I readily admit that there are several scanned maps which I examined during my doctoral studies that I’ve never seen, let alone handled, in person. This is not a criticism: the digitisation of maps, and of collections more generally, has opened up and made archival materials widely accessible. In a context of declining funding for humanities and social science research across the board (a serious problem for map history but a potential future *Forum* topic), an obvious practical benefit is it helps attenuate the impact incurred by significant costs of travel and accommodation which can limit access to primary sources for early career and even established scholars. Digitisation is, of course, not without its problems, and it should be acknowledged that the ‘shift in power in digital archives is not from archivists to researchers, but from archivists to algorithms.’^6^ The unevenness behind which map collections are, or have the means to be, digitised can also shape what maps, mapping projects, or mapmakers get researched and obscure those which do not have a digital presence.

That said, it remains quite difficult to shake off the notion that ‘good’ map history still requires scholars and curators to engage with the material in an experiential sense, whether unfolding a specific map by hand, turning the pages of an atlas, or, in other cases, going through the physical papers or collection of a mapmaker or mapping project at large, appreciating the depth of colouring, fine details, annotations and errors, paper texture, and other material aspects in a way which a digital image does not allow. To give an anecdotal example, a student at King’s College London approached me for advice on doing a dissertation project on Charles Booth’s London Poverty maps and I finished our discussion by instinctively telling the student ‘I expect you’ll be contacting the archivist at the London School of Economics (LSE) to see the maps in person even though they’re digitised.’ The context is important: LSE is accessible for a student at King’s, as both are in London, and I assumed the student would understand that seeing and even handling the maps would help them make sense of why it was made, what its purpose was and how it was used. However, in a shifting landscape where the line between the non-digital and digital is blurred for many, the distinction between the material and the digitised map may no longer be clear or even relevant.^7^

## Making Map History Digital

The League of Nations map collection was the working collection of one of the first truly international or intergovernmental organisations founded in the aftermath of the First World War. The geographical department, as the collection came to be known, was part of the League’s Library and, by the late 1930s, claimed to have one of the most complete collections of modern maps in the world (c. 28,000 sheets). The collection was transferred to the newly founded UN in 1946, although the LON map collection remains separately catalogued from the UN map collection in Geneva today. Unsurprisingly, the geographical department had wide international coverage, with a particular distinctiveness in its focus on thematic mapping reflecting the League’s immediate concerns: maps covering ethnographic and linguistic, health and social, and Mandates and territorial questions. In addition to collecting maps produced by national governments and private entities, the geographical department produced maps for reports and other publications.

In 2017, the UN Library & Archives at Geneva commenced the *Total Digital Access to the League of Nations Project* (LONTAD), a five-year project to digitise and make available online the entire League of Nations archives, including the map collection. To give a sense of the scale of the project, 14.2 million pages of material was digitised by more than thirty people, amounting to over 220 terabytes of data.^8^ The rationale for the LONTAD project was openness, accessibility, and preservation: the Library and Archives distinguishes between physical and digital preservation, with the former focused on restoration and the latter concerned with ‘guaranteeing’ access and futureproofing its collection. In other words, the LONTAD project was like most other digitisation projects.

Less typical, however, is the policy which followed digitisation. If someone requests to see the maps—or any digitised LON material—they are informed that the materials are no longer available for consultation for reasons of preservation. Map historians who pre-dominantly work on twentieth-century mapping are not often forced to contend with the idea that the maps we study need to be conserved. I certainly recognise that unique maps may require stricter preservation protocols, but the sorts of topographic map series held in the League’s collection are widely found in map libraries across the world. Perhaps the assumption that the maps are common helps explain the UN’s approach: these maps are physically accessible elsewhere so there is no need to make them available in Geneva.

Paradoxically, this post-digitisation policy heightens awareness of the map’s materiality as fragile and ephemeral, and whose long-term survival can only be guaranteed by converting the object into a string of data, observable through an interface. Map scholars are then left with a Kafkaesque situation where a researcher could be sitting in the reading room in Geneva with an open laptop and examining the LON maps through the digital portal, which takes time to get used to. The issue is that the League’s mapping—and its place in the *history of* mapping—becomes a story only mediated through the disaggregated digital object, displacing maps from the ‘collection.’ Loss of the original context, as well as access to the physical document, can cause both scholars and curators to lose sight of how groups of maps or collections, and not just individual maps, shape institutions such as the League.

## Picturing the Back (End)

Preservation is generally a good thing, and map history has a limited future without maps from the peoples, places, and times under study. A concern with digitisation is that it opens the door to consolidation (removing duplicate maps held internally, or available elsewhere), or selling and/or dispersing significant parts or entire collections.^9^ Avoiding duplication can make sense when done right, but programmes to consolidate or disperse parts of a collection can be driven by external pressures which see digitisation as a replacement rather than a complementary process. The UN is not immune to these pressures, as it struggles with a funding crisis that has affected its Library and Archives.^10^ Given the footprint that maps take up in storage, they are potentially at higher risk of deaccession than other paper objects when space becomes a premium. Of more immediate concern with digitisation is the user friendliness of the interface and how useful the metadata is, both of which can impact doing good map history.^11^ In the case of the LON archives, metadata and descriptions provide little information about each item’s acquisition, use, or context, although this is perhaps understandable given the relatively short five-year period of a project to digitise over 14 million items, and detailed descriptions take time.

The LONTAD project raises a more important question about the role of cartographic expertise in shaping how digitisation happens. One issue that casts some doubt on the level of subject-specific expertise involved in scanning and digitising maps is the assumption that it’s unnecessary to scan the back of maps with no print matter on them, even though, in my experience, working collections such as the LON are likely to have interesting annotations or notes. This is not unique to the LON digitised collection but my request to see the physical maps to check for annotations or notes was not deemed a justifiable reason to do so, although it clearly took the UN Library and Archives staff by surprise. This not a criticism of their decisions on digitisation or physical access; significant institutional archives such as the UN will be staffed by a larger proportion of generalists than specialists to look after a diverse collection that includes maps, books, reports, papers, photographs, posters, and other materials.

Nevertheless, a lack of subject-expert involvement in digitisation projects which include maps does have implications for what ‘gets left out’ and can influence the ways in which map history gets done. My argument about the importance of examining the reverse of the LON’s maps may have been over-stated and, indeed, not successful. Nonetheless, the difference between the digital and material objects in this collection speaks to the murkier issue of the backend of digitisation programmes and digital interfaces which gets hidden away from users. Digitisation is a powerful (and in some cases, deeply political) process of organising and building a new collection, even if institutions claim that the digital index simply mirrors how the physical collection has been catalogued.^12^ And while I’m sure many archives see themselves as transparent, the openness and accessibility of an online catalogue and user friendly interface can obscure the decisions behind what gets—and what does not get—scanned or preserved, the reasons for how a collection is organised and hierarchised, and how lines of code and algorithms can shape what and how we research.^13^

I do not suggest that the digital will entirely replace the material when it comes to doing map history any time soon (except for the LON collection—for now). Map history remains primarily a world of paper (and many other materials) to which anyone who has attended major conferences such as the ICHC and ISHMap or read *Imago Mundi* can attest. And the tradition of physically visiting a collection or library to research maps, globes, and atlases—to experience and understand the material—will still matter, especially to doctoral and early career researchers in map history.

That said, I’m also fully aware that I make this point from a place of privilege, with research funding that allows me to visit collections around the world and to cultivate the kinds of questions, answers, and approaches that shape the field of map studies based on consultation of physical maps and the papers connected to them. I am doubly fortunate to work in a city that offers low cost and generally open access to several of the world’s most comprehensive map collections, including the British Library and the Royal Geographical Society. Digitisation, therefore, can be a leveller that enables researchers without the same privileges or access to produce excellent work from diverse perspectives that will help advance the field. It is possible that an intellectual and methodological divide may emerge between scholars that have access to physical collections and those that use digital collections only. But given the trend of declining funding for humanities and social science research in many countries and institutions, map history mediated through the digital may become accepted practice for most scholars.

As it does, scholars, journals, publishers, and institutions must recognise, be open to, and find ways to support the generations of potential future map historians for whom doing research virtually and remotely using digital archives is the norm rather than the exception. One way would be to provide more time for research, recognising that become- or born-digital research still requires a high level of non-financial investment. There is also scope for more innovation in how scholars and curators collaborate, using evolving technologies such as virtual reality to enable remote ‘visits,’ particularly to allow remote users to consult about aspects, such as undigitised versos of maps, that are not available for virtual consultation.^14^ The new norm may simply encourage or require more openness in sharing notes, findings and experiences between those who have worked with physical materials and those who have not. In the longer run perhaps, scholars and curators will have to reconsider whether materiality remains necessary for map historical research. What is important for the future is that we critically attend to these changes in how we do map history.
